# CCR4‐NOT subunit CCF‐1/CNOT7 promotes transcriptional activation to multiple stress responses in *Caenorhabditis elegans*

**DOI:** 10.1111/acel.13795

**Published:** 2023-02-16

**Authors:** Hadi Tabarraei, Brandon M. Waddell, Kelly Raymond, Sydney M. Murray, Ying Wang, Keith P. Choe, Cheng‐Wei Wu

**Affiliations:** ^1^ Department of Veterinary Biomedical Sciences, Western College of Veterinary Medicine University of Saskatchewan Saskatoon Saskatchewan Canada; ^2^ Department of Biology and Genetics Institute University of Florida Gainesville Florida USA; ^3^ Toxicology Centre University of Saskatchewan Saskatoon Saskatchewan Canada; ^4^ Department of Biochemistry, Microbiology and Immunology, College of Medicine University of Saskatchewan Saskatoon Saskatchewan Canada

**Keywords:** aging, *C. elegans*, longevity, oxidative stress, stress response

## Abstract

CCR4‐NOT is a versatile eukaryotic protein complex that controls multiple steps in gene expression regulation from synthesis to decay. In yeast, CCR4‐NOT has been implicated in stress response regulation, though this function in other organisms remains unclear. In a genome‐wide RNAi screen, we identified a subunit of the CCR4‐NOT complex, *ccf‐1*, as a requirement for the *C. elegans* transcriptional response to cadmium and acrylamide stress. Using whole‐transcriptome RNA sequencing, we show that the knockdown of *ccf‐1* attenuates the activation of a broad range of stress‐protective genes in response to cadmium and acrylamide, including those encoding heat shock proteins and xenobiotic detoxification. Consistently, survival assays show that the knockdown of *ccf‐1* decreases *C. elegans* stress resistance and normal lifespan. A yeast 2‐hybrid screen using a CCF‐1 bait identified the homeobox transcription factor PAL‐1 as a physical interactor. Knockdown of *pal‐1* inhibits the activation of *ccf‐1* dependent stress genes and reduces *C. elegans* stress resistance. Gene expression analysis reveals that knockdown of *ccf‐1* and *pal‐1* attenuates the activation of *elt‐2* and *elt‐3* under stress that encode master transcriptional co‐regulators of stress response in the *C. elegans*, and that overexpression of ELT‐2 can suppress *ccf‐1*'s requirement for gene transcription in a stress‐dependent manner. Our findings reveal a new role for CCR4‐NOT in the environmental stress response and define its role in stress resistance and longevity in *C. elegans*.

## Graphical abstract

This study reveals a new role for the CCR4‐NOT complex in transcriptome regulation in response to environmental stress using the *C. elegans* model. CCF‐1/CNOT7 encoding the deadenylase subunit of the complex is required for activation of xenobiotic detoxification genes in response to stress, which in turn is critical for enhancing whole‐organism stress resistance as well as maintaining longevity.

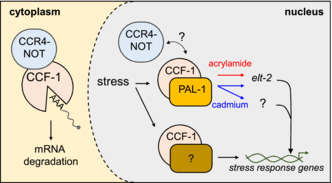

**Abbreviations**
ARP‐2/3: Actin‐related protein
ARX: Actin‐related protein complex
ATF‐7: Activating transcription factor
CDX: Caudal‐related homeobox 1
CCF‐1: Carbon catabolite repression associated factor
CCR4‐NOT: Carbon catabolite repression 4 – negative on TATA‐less
CDC‐42: Cell division cycle related
DAF‐16: Dauer formation
ELT‐2: Erythroid‐like transcription factor
EV: Empty vector
GPDH‐1: Glycerol‐3‐phosphate dehydrogenase
GST: Glutathione S‐transferase
HIZR‐1: High zinc activated nuclear receptor
HLH‐1: Helix loop helix
HSF‐1: Heat shock factor
LET‐711: Lethal
MTF‐1: Metal responsive transcription factor
NHR‐46: Nuclear hormone receptor
NTL‐2: Negative on TATA‐less‐like
NUMR‐1: Nuclear localized metal response
PAL‐1: Posterior Alae
RNR: Ribonucleotide reductase
RPL‐2: Ribosomal protein large subunit
RRF‐3: RNA‐dependent RNA polymerase family
SKN‐1: Skinhead
STA‐2: Signal Transducer and Activator
XREP‐4: Xenobiotics response pathways
Y2H: Yeast 2‐hybrid

## 1 INTRODUCTION

In response to environmental stress, organisms can mount various adaptive responses to promote survival. At the cellular level, a major form of adaptive response is the transcriptional activation of genes that serve to prevent or repair cellular damage during periods of stress. Examples of such responses include the induction of xenobiotic detoxification and antioxidant genes to alleviate oxidative stress or the activation of chaperone genes functioning in protein folding to maintain proteostasis under heat shock (An et al., 2005; Sarge et al., 1993). In the nematode *Caenorhabditis elegans*, various transcriptional pathways that regulate environmental stress response have also promoted longevity (Morley & Morimoto, 2004; Tullet et al., 2008). Consistently, impaired stress response contributes to aging in *C. elegans* and has also been linked to various human diseases (Hekimi et al., 2011; Morley & Morimoto, 2004; Tullet et al., 2008). As such, uncovering new insights toward understanding molecular mechanisms of stress response pathways are of widespread interest.

Our previous work in *C. elegans* showed that exposure to the heavy metal cadmium strongly activates a gene named *numr‐1* (nuclear‐localized metal response), which encodes a putative RNA binding protein required for cadmium resistance (Tvermoes et al., 2010; Wu et al., 2019). The mechanism of cadmium‐induced *numr‐1* activation appears to be distinct from the hallmark induction of metallothionein‐encoding genes that maintain homeostasis by scavenging and removing xenobiotic metals (Sabolić et al., 2010). Cadmium activation of *numr‐1* partially signals through the transcription factor HSF‐1 (Heat Shock Factor), whereas metallothionein gene activation in *C. elegans* requires the DAF‐16 (Dauer Formation)/FoxO transcription factor (Barsyte et al., 2002; Wu et al., 2019). Given that cadmium is known to induce many types of cellular alterations including oxidative stress, DNA damage, and RNA splicing disruption, it is not surprising that multiple stress response pathways are simultaneously activated to promote cellular resistance (Bertin & Averbeck, 2006; Chomyshen et al., 2022). Additionally, cadmium activates the expression of genes encoding various detoxification enzymes involved in glutathione metabolism that is controlled by the SKN‐1 (SkiNhead)/Nrf2 transcription factor (An et al., 2005). A recent study has also implicated the role of the nuclear hormone receptor HIZR‐1 (High Zinc activated nuclear Receptor) in regulating the expression of cadmium and zinc‐responsive genes in cooperation with the mediator transcriptional complex (Shomer et al., 2019). The involvement of multiple transcription factors in response to metal stress in *C. elegans* may also reflect the lack of the MTF‐1 (Metal responsive Transcription Factor) gene in nematodes that serves as the conserved regulator of metal‐induced transcriptional response in fly, fish, and mammals (Günther et al., 2012). While the requirement of multiple transcription factors involved in stress responses is well recognized, less known are co‐regulators that assist in these transcriptional activation events.

In this study, we used *numr‐1* as a stress marker to identify a new role for the CCR4‐NOT (Carbon Catabolite Repression 4 – Negative On TATA‐less) complex in regulating *C. elegans* transcriptional response to multiple environmental stress. The CCR4‐NOT complex was originally identified as an eukaryotic deadenylase that post‐transcriptionally regulates mRNA stability through poly‐A tail removal (Collart, 2016; Nousch et al., 2013). Subsequent studies in yeast have also revealed a role for the CCR4‐NOT in transcriptional initiation through interactions with RNA polymerase II, implicating a broad regulatory function of this complex in mRNA synthesis and decay (Collart, 2016). A direct role of the CCR4‐NOT complex in stress regulation has also been recently reported in yeast (Mulder et al., 2005). Here, we demonstrate that the *C. elegans* CCR4‐NOT deadenylase subunit *ccf‐1* (CCR4‐associated factor) is broadly required for stress‐induced transcriptional programming in *C. elegans* in response to cadmium and acrylamide. Our results show that *ccf‐1* is required for a normal lifespan along with stress resistance, and its knockdown also shortens the longevity of multiple long‐lived mutants. Through the yeast 2‐hybrid (Y2H) system, we identified the homeobox PAL‐1 (Posterior Alae) transcription factor as a novel physical interactor of CCF‐1 and uncovered a previously undescribed role for this protein in the regulation of stress response genes in *C. elegans*. Overall, this study provides new insights into genetic regulators of transcriptional response and resistance to environmental stress in *C. elegans*.

## 2 RESULTS

### 2.1 Components of the CCR4‐NOT complex are necessary for the cadmium stress response

**FIGURE 1:**
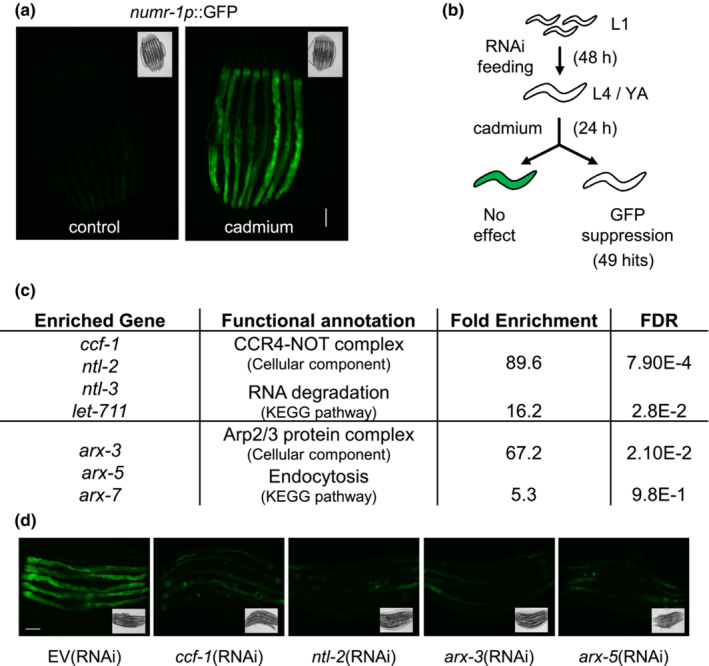
Genome‐wide RNAi screen reveals a requirement for genes encoding the CCR4‐NOT complex for cadmium‐induced activation of *numr‐1p*::GFP. (a) Representative fluorescence and brightfield micrographs of worms expressing *numr‐1p*::GFP under control and cadmium conditions. (b) Workflow of genome‐wide RNAi screen. (c) DAVID enrichment of dsRNA clones that inhibited cadmium‐induced *numr‐1p*::GFP expression. (d) Representative fluorescent and brightfield micrographs of worms fed dsRNA resulting in the inhibition of cadmium‐induced expression of *numr‐1p*::GFP. The scale bar is 100 μm.

We previously showed that the cadmium inducible *numr‐1* gene encodes as a putative RNA binding protein that influences RNA splicing in *C. elegans* (Wu et al., 2019). To screen for regulators of this stress response, we performed a genome‐wide RNAi screen using a *numr‐1p*::GFP transcriptional reporter to identify genes that when knocked down inhibit the induction of *numr‐1p*::GFP after cadmium exposure (Figure 1a,b). We identified 49 genes that when knocked down reduced *numr‐1p*::GFP after cadmium and had minimal effect on *C. elegans* development. Enrichment analysis of these 49 genes revealed functional annotation to two protein complexes, the CCR4‐NOT complex and the Arp2/3 (Actin‐related protein) complex (Figure 1c; Table S1). Fluorescence levels of *numr‐1p*::GFP after cadmium exposure were highly suppressed when four genes encoding subunits of the CCR4‐NOT complex and three genes encoding subunits of the Arp2/3 complex were knocked down by RNAi (Figure 1c). Representative fluorescent micrographs on the effects of *ccf‐1*, *ntl‐2*, *arx‐3*, and *arx‐5* RNAi on *numr‐1p*::GFP expression in cadmium are shown (Figure 1d). The CCR4‐NOT complex is well characterized for its role as an mRNA deadenylase that facilitates poly‐A tail removal, a cellular process that decreases transcript stability that is highly conserved in yeast, worm, and humans (Nousch et al., 2013; Shirai et al., 2014). However, emerging evidence in yeast suggests that the CCR4‐NOT complex is also involved in transcriptional regulation, though this molecular mechanism is less understood (Kruk et al., 2011; Mulder et al., 2005). As such, we focused on characterizing the role of the CCR4‐NOT complex in the regulation of stress‐induced transcription in this study.

**FIGURE 2:**
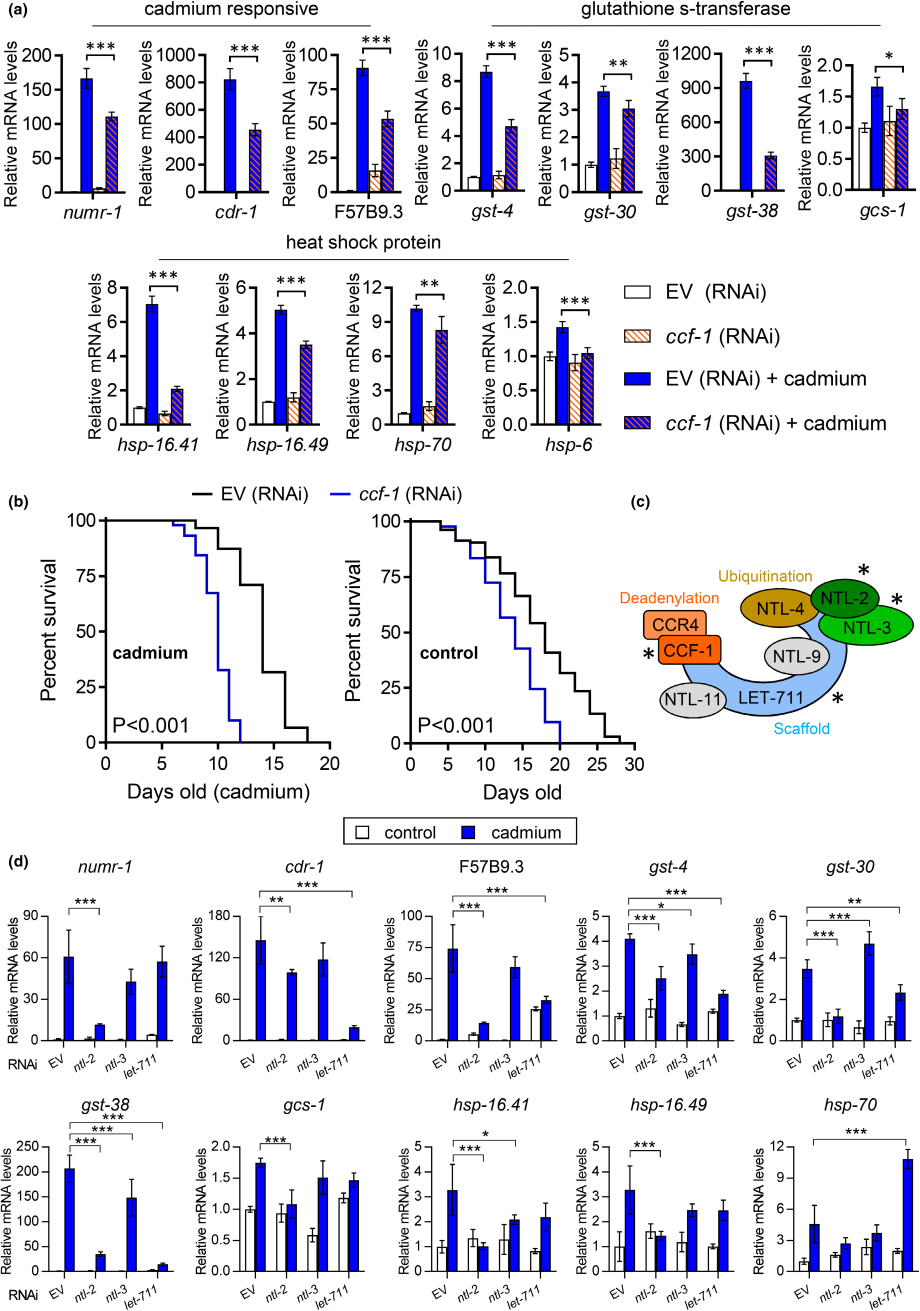
Components of the CCR4‐NOT complex regulate the cadmium stress response. (a) Relative expression of cadmium‐responsive genes in N2 worms fed with EV or *ccf‐1* RNAi under basal conditions or after exposure to cadmium. (b) Cadmium survival and lifespan curves of N2 worms fed with EV or *ccf‐1* RNAi, *p* < 0.001 as determined by the log‐rank test. Results for trial 1 are shown for cadmium survival and trial 2 for lifespan curve, full results, and statistics for all trials are presented in Table S3. (c) Components of the *C. elegans* CCR4‐NOT complex with * indicating genes identified to be required for cadmium‐induced *numr‐1p*::GFP from the RNAi screen. (d) Fold change in expression of cadmium‐responsive genes in N2 worms fed with EV, *ntl‐2*, *ntl‐3*, or *let‐711* under basal conditions or after exposure to cadmium. For a and d, bar graphs display means and error bars indicate the standard deviation of *N* = 4 samples. *p < 0.05, **p < 0.01, and ***p < 0.001 compared to EV (RNAi) + cadmium as determined by two‐way ANOVA with Holm‐Sidak post hoc tests.

To determine if the CCR4‐NOT complex is required for the activation of a broad range of cadmium inducible genes, we used RNAi to knock down the *ccf‐1*/*CNOT7* gene that encodes the deadenylase subunit of the CCR4‐NOT complex (Nousch et al., 2013). Using qPCR, we found that expressions of various classes of cadmium‐induced genes are significantly reduced in worms fed with *ccf‐1* RNAi as compared to the empty vector (EV) control after cadmium exposure, including those encoding glutathione s‐transferase (*gst*) and heat shock protein (*hsp*) genes (Figure 2a). Consistent with *ccf‐1* functioning as a requirement for the cadmium stress response, the knockdown of *ccf‐1* reduced *C. elegans* cadmium resistance; meanwhile, the knockdown of *ccf‐1* also significantly reduced *C. elegans* lifespan (Figure 2b). To determine if other subunits of the CCR4‐NOT complex are also required for the activation of cadmium‐responsive genes (Figure 2c), we knocked down three other genes identified from the genome‐wide RNAi screen that encode subunits of the CCR4‐NOT complex and performed qPCR analysis. RNAi knockdown of *ntl‐2* (NOT‐like)/*CNOT2* suppressed the activation of 9 out of 10 cadmium‐induced genes tested, whereas knockdown of *ntl‐3*/*CNOT3* and *let‐711* (LEThal)/*CNOT1* suppressed the activation of 2 and 5 out of 10 cadmium‐induced genes, respectively (Figure 2d). The relative variance in gene expression levels may suggest a differential requirement of each CCR4‐NOT subunit in regulating cadmium induced transcriptional response. It is also possible that the difference may be due to varying degrees of RNAi penetrance between the dsRNA clones. Although RNAi depletion of *ccf‐1*, *ntl‐2*, and *ntl‐3* all led to F_1_ embryonic lethality and *let‐711* RNAi led to P_0_ L3/L4 arrest (data not shown). Overall, these data suggest that subunits of the CCR4‐NOT complex are required for *C. elegans* transcriptional response to cadmium stress and that the *ccf‐1* deadenylase subunit is essential for normal lifespan and cadmium survival.

### 2.2 *ccf‐1* is required for the transcriptional response activated by multiple stressors

**FIGURE 3:**
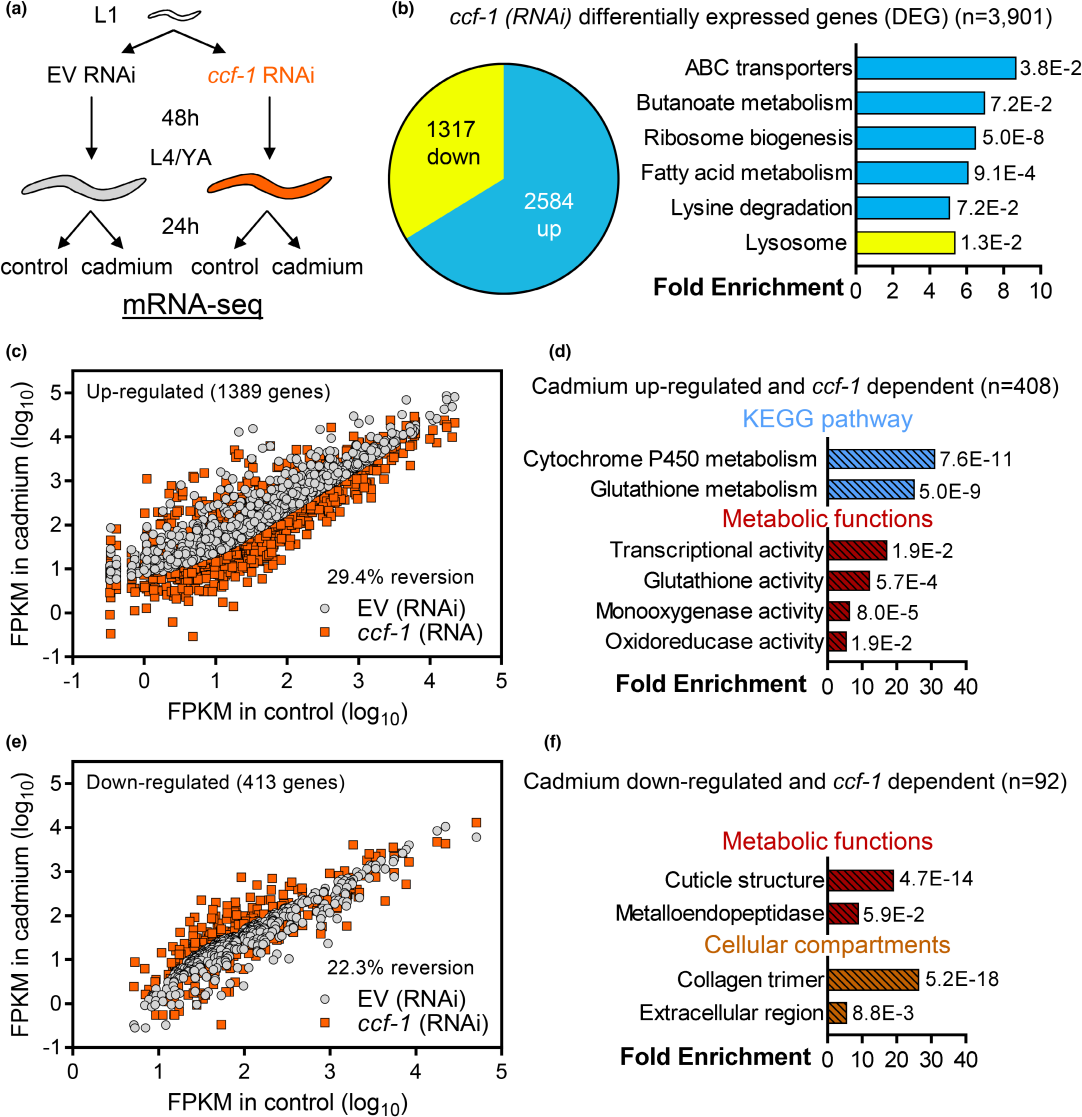
Whole‐transcriptome sequencing reveals *ccf‐1* is broadly required for cadmium‐induced gene expression. (a) Experimental workflow used for preparing RNA sequencing samples. (b) Pie chart illustrating the number of genes significantly up‐regulated or down‐regulated in N2 worms by >2‐fold after *ccf‐1* (RNAi) compared to EV. The bar graph illustrates DAVID analysis of KEGG pathways significantly enriched for genes up‐regulated (blue) or down‐regulated (yellow) after *ccf‐1* (RNAi) compared to EV in control conditions. Fold enrichment and FDR values are shown for each KEGG pathway. (c) Scatter‐plot of 1389 cadmium up‐regulated genes (>2‐fold) in N2 worms fed with EV compared to their corresponding expression in N2 worms fed with *ccf‐1* (RNAi). (d) Enriched KEGG pathways and metabolic functions of 408 *ccf‐1*‐dependent cadmium up‐regulated genes. (e) Scatter‐plot of 413 cadmium down‐regulated genes (>2‐fold) in N2 worms fed with EV compared to their corresponding expression in N2 worms fed with *ccf‐1* (RNAi). (f) Enriched metabolic functions and cellular compartments of 92 *ccf‐1*‐dependent cadmium down‐regulated genes. Reversion % in c and e represent % of genes whose expression was altered by >2‐fold in the opposing direction by *ccf‐1* (RNAi) compared to EV.

Given that knockdown of *ccf‐1* led to a consistent reduction in the expression of select cadmium inducible genes, we next examined the effect of *ccf‐1* knockdown on the whole transcriptome before and after cadmium exposure using RNA sequencing (Figure 3a; Table S2). Knockdown of *ccf‐1* under basal conditions led to differential expression of 3901 genes by more than twofold, with the majority of these genes up‐regulated (Figure 3b). Gene up‐regulation in response to *ccf‐1* knockdown in the absence of stress was also observed for select cadmium inducible genes (*numr‐1*, F57B9.3) in our qPCR data (Figure 2a), and is consistent with the known deadenylase function of CCF‐1 where its knockdown results in the retention of mRNA poly‐A tail that increases transcript stability (Nousch et al., 2013). Enrichment analysis revealed that genes up‐regulated after *ccf‐1* knockdown cluster to KEGG pathways including ABC transporters, ribosome biogenesis, and lipid metabolism, whereas genes down‐regulated by *ccf‐1* RNAi cluster to the lysosome pathway (Figure 3b).

Next, we examined the effects of *ccf‐1* knockdown on genes that are differentially regulated in cadmium by more than twofold. Of the 1389 cadmium up‐regulated genes, 408 were found to be suppressed by >2‐fold in *ccf‐1* knocked‐down worms indicating their dependence on *ccf‐1* for cadmium‐induced gene expression (Figure 3c). The 29.4% (408/1389) reversion effect (>2‐fold change) of cadmium‐induced genes by *ccf‐1* RNAi is 6.25‐fold greater than the 4.7% reversion effect observed across genes whose expression is unaffected by cadmium (Figure S1a). Enrichment analysis of the 408 *ccf‐1*‐dependent cadmium genes reveals that they cluster to xenobiotic detoxification pathways and metabolic functions including cytochrome P450 metabolism and glutathione activity (Figure 3d). To determine if the requirement for *ccf‐1* in buffering transcript levels is specific to stress, we found that 64 out of 1389 cadmium up‐regulated genes are also dependent on *ccf‐1* under control conditions. However, these 64 genes do not enrich detoxification pathways suggesting that *ccf‐1* is specifically required for xenobiotic gene expression under stress. We next examined the 413 cadmium down‐regulated genes and found that 92 were *ccf‐1* dependent (22.3% reversion, Figure 3e; vs. 10.8% reversion in non‐cadmium‐responsive genes, Figure S1a); these 92 genes enriched to cuticle structure and collagen/extracellular maintenance (Figure 3f; no enrichment toward KEGG pathway was found). Under control conditions, 26 out of 413 cadmium down‐regulated genes were *ccf‐1* dependent, however, these genes do not enrich any specific pathway. Together, these results demonstrate that *ccf‐1* is an essential factor in regulating a transcriptional response upon cadmium stress exposure.

**FIGURE 4:**
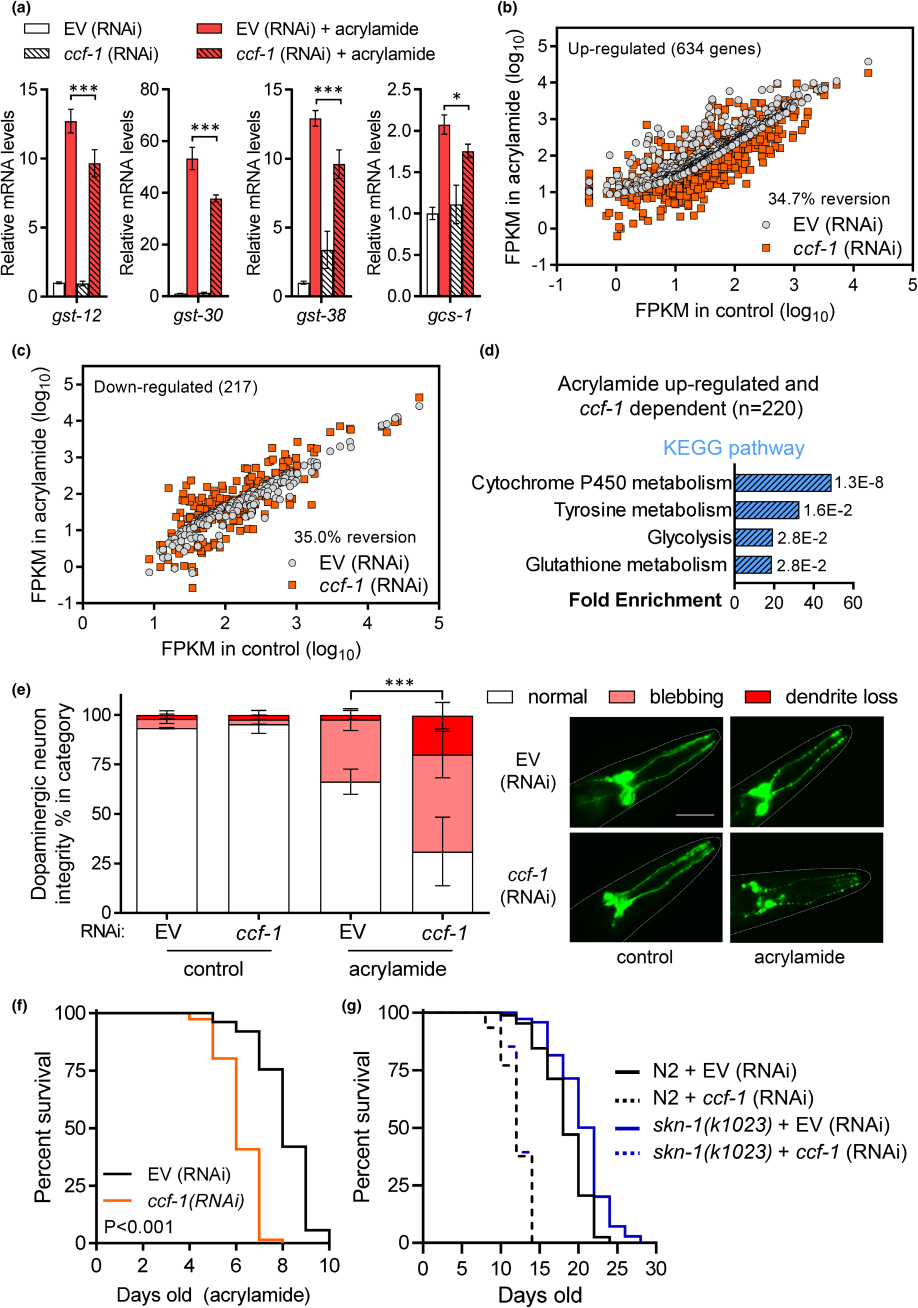
Depletion of *ccf‐1* diminishes *C. elegans* resistance to acrylamide. (a) Relative expression of *gst* genes and *gcs‐1* in worms fed with EV or *ccf‐1* (RNAi) under basal conditions, or after exposure to acrylamide. Bar graphs display means and error bars indicate the standard deviation of *N* = 4 samples. *p < 0.05 and ***p < 0.001 compared to EV (RNAi) + acrylamide as determined by two‐way ANOVA with Holm‐Sidak post hoc tests. Scatter‐plot of (b) 634 acrylamide up‐regulated genes and (c) 217 acrylamide down‐regulated genes in N2 worms fed with EV compared to their corresponding gene expression in N2 worms fed with *ccf‐1* (RNAi). Reversion % in b and c represent % of genes whose expression was altered by >2‐fold in the opposing direction by *ccf‐1* (RNAi) compared to EV. (d) Enriched KEGG pathways of the 220 *ccf‐1* dependent acrylamide up‐regulated genes. (e) Dopaminergic neuron integrity of worms fed with EV or *ccf‐1* (RNAi) under basal conditions, or treated with acrylamide. Results are combined from three trials of *N* = 15 worms per condition scored in each trial. Representative fluorescent micrographs of control or acrylamide‐treated worms expressing the *dat‐1p*::GFP reporter fed with EV or *ccf‐1* (RNAi), scale bar is 50 μm. Error bars indicate the standard error of the mean, ***p < 0.001 as determined by the Chi‐square test. (f) Acrylamide survival curve of N2 worms fed with EV or *ccf‐1* (RNAi), *p* < 0.001 as determined by the log‐rank test. Results for trial 2 are shown for acrylamide survival, full results, and statistics for all trials are presented in Table S3. (g) Lifespan curves of N2 and *skn‐1(k1023)* gain of function worms fed with EV or *ccf‐1* (RNAi). Results for trial 1 are shown with full results and statistics for all trials are presented in Table S3.

As *ccf‐1* is required for the expression of antioxidant genes in response to cadmium, we then examined if *ccf‐1* is required for mounting a stress response to acrylamide, which is a potent inducer of oxidative stress (Wu et al., 2017). Using qPCR, we found that the knockdown of *ccf‐1* reduced the activation glutathione related antioxidant genes after acrylamide exposure including *gst‐12*, *gst‐30*, *gst‐38*, and *gcs‐1* (Figure 4a). Next, we expanded this analysis to the whole transcriptome by using RNA sequencing and found that the knockdown of *ccf‐1* showed a 34.7% and 35.0% reversion rate for acrylamide up and down‐regulated genes respectively (Figure 4b,c), both are higher than the *ccf‐1* knockdown reversion rates of 6.3% (>2‐fold down‐regulated) and 8.1% (>2‐fold up‐regulated) observed for genes that do not respond to acrylamide (Figure S1b). Of the 634 acrylamide up‐regulated genes, 220 were found to be *ccf‐1* dependent and they enrich cytochrome P450 and glutathione metabolism pathways (Figure 4d). Under control conditions, 45 out of 634 acrylamide up‐regulated genes were found to be *ccf‐1* dependent, however, these genes do not enrich xenobiotic detoxification pathways. This pattern is similar to that observed under cadmium where *ccf‐1* is required for the expression of detoxification genes only when worms are under stress. These results support a general requirement for *ccf‐1* in the transcriptome regulation of the acrylamide stress response. Consistent with this conclusion, the knockdown of *ccf‐1* exacerbated acrylamide's neurotoxicity toward dopaminergic neurons (Figure 4e) and reduced *C. elegans* survival to acrylamide (Figure 4f). In addition to acrylamide, we show that the knockdown of *ccf‐1* attenuates the activation of *gpdh‐1p*::GFP in response to osmotic stress (Figure S1c), suggesting a broad role for *ccf‐1* in regulating multiple types of stress responses beyond acrylamide and cadmium.

Overall, these results demonstrate a transcriptome‐wide dependency on *ccf‐1* for the expression of detoxification genes in response to stress, and that these same stress genes generally do not require *ccf‐1* for their expression under control conditions.

### 2.3 Stress‐resistant long‐lived mutants required *ccf‐1* for longevity

Transcriptomic analysis revealed that *ccf‐1* is required for the expression of various stress‐responsive genes controlled by the transcription factors SKN‐1 and HSF‐1 that also promote longevity in *C. elegans* (Baird et al., 2014; Tullet et al., 2008). To test whether *ccf‐1* is required for SKN‐1  and HSF‐1 dependent longevity, we knocked down *ccf‐1* in the *skn‐1(k1023)* gain of function mutant and a full‐length *hsf‐1(FL)* overexpression strain (Baird et al., 2014; Tang & Choe, 2015). Knockdown of *ccf‐1* reduces wildtype lifespan (Figure 1b) and we found that it completely suppressed the longevity of the *skn‐1(k1023)* mutant but only partially suppressed the longevity of *hsf‐1(FL)* worms (Figure 4g and Figure S2a). On EV RNAi, *hsf‐1(FL)* extended median lifespan by +35% compared to N2, but this extension is reduced to +16% when fed with *ccf‐1* RNAi. The *skn‐1* gene also acts downstream of the insulin signaling pathway to promote the longevity of the insulin receptor *daf‐2* mutant (Tullet et al., 2008). Compared to wildtype, the *daf‐2(e1370)* mutant was able to extend its lifespan while fed with *ccf‐1* RNAi to a similar extent as that of EV RNAi (Figure S2b; median lifespan +133% in EV RNAi vs +119% in *ccf‐1* RNAi). Overall, these results show that *ccf‐1* is completely required for the longevity of the *skn‐1(k1023)* mutant, but only partially required for longevity in the *hsf‐1(FL)* and *daf‐2(e1370)* mutant.

### 2.4 CCF‐1 localizes to the nucleus and interacts with the PAL‐1 transcription factor

To gain insights into how *ccf‐1* influences stress‐responsive genes, we generated a strain of *C. elegans* expressing an integrated multicopy *ccf‐1p*::CCF‐1::GFP transgene to test if CCF‐1 protein expression is influenced by stress. CCF‐1::GFP is expressed in multiple tissues and intestinal nuclear CCF‐1::GFP can be observed in ~50% of the animals under basal conditions (Figure S3a). Exposure to cadmium or acrylamide did not alter the relative nuclear distribution or total fluorescence of CCF‐1::GFP, suggesting that the expression and localization of the CCF‐1 protein are not influenced by stress (Figure S3b–d). However, the nuclear presence of CCF‐1 suggests potential alternative functions other than its characterized cytoplasmic deadenylase role.

Given that knockdown of *ccf‐1* reduced lifespan and stress resistance (Figure 2b), we next tested how these parameters are affected in worms expressing the multicopy CCF‐1::GFP transgene where *ccf‐1* is overexpressed (Figure S3e). We found that in the CCF‐1::GFP strain, there was no extension to normal lifespan and stress resistance compared to the wildtype (Figure S3f), suggesting that overexpression of *ccf‐1* alone is not sufficient in enhancing longevity and stress resistance.

**FIGURE 5:**
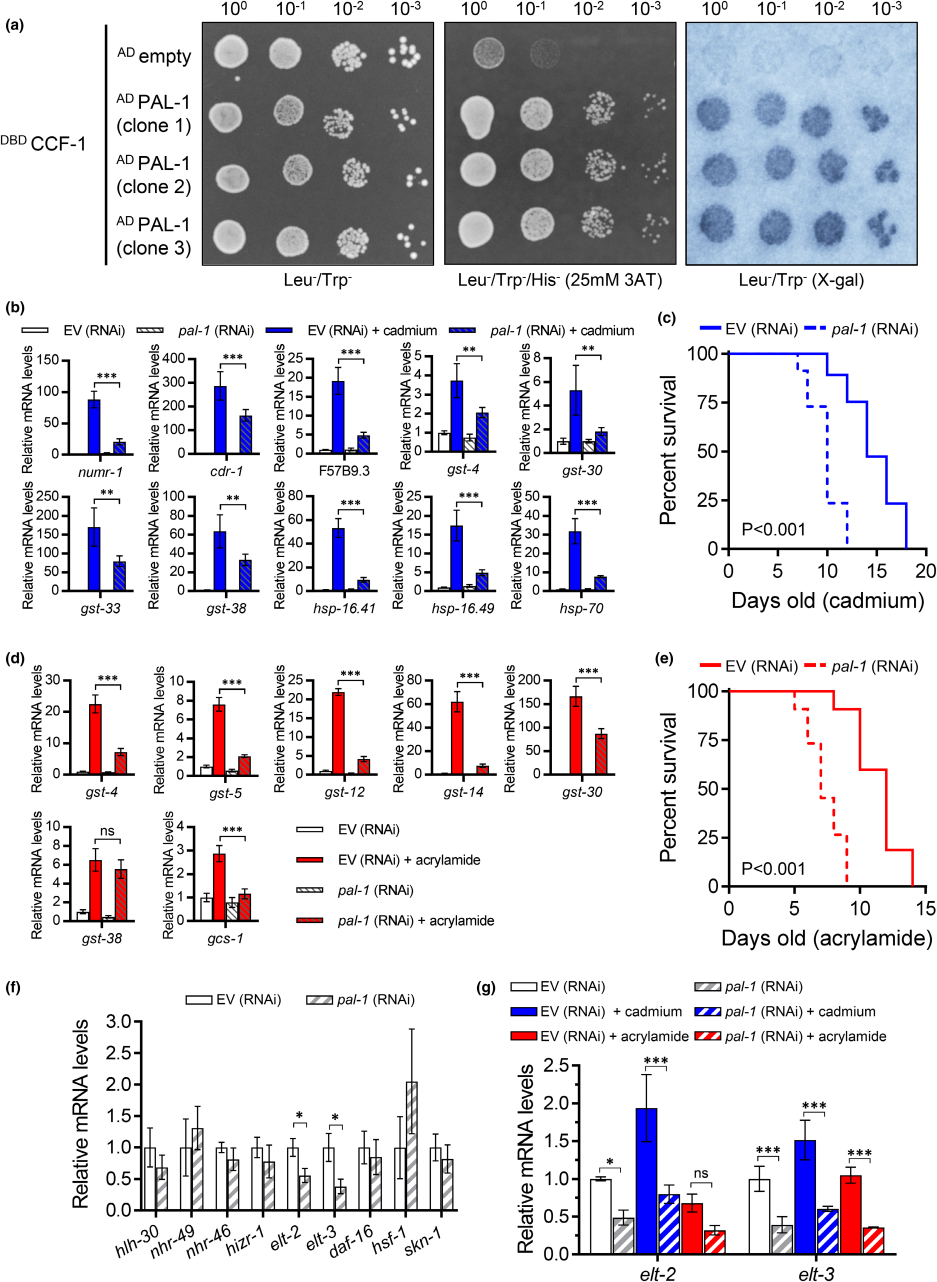
PAL‐1 interacts with CCF‐1 and regulates stress‐responsive genes. (a) Y2H interactions between CCF‐1 (bait) and three independent clones of PAL‐1 (prey) on the control Leu^−^/Trp^−^ plate, Leu^−^/Trp^−^/His^−^/25 mM 3AT selection plate, and Leu^−^/Trp^−^ X‐gal assay. The X‐gal image is converted from grayscale to blue. (b) Relative expression of cadmium response genes under control or cadmium exposure in *rrf‐3(pk1426)* mutant worms fed with EV or *pal‐1* (RNAi). (c) Cadmium survival of *rrf‐3(pk1426)* worms fed with EV or *pal‐1* (RNAi). (d) Relative expression of acrylamide response genes under control or acrylamide exposure in *rrf‐3(pk1426)* worms fed with EV or *pal‐1* (RNAi). (e) Acrylamide survival of *rrf‐3(pk1426)* worms fed with EV or *pal‐1* (RNAi). Trial 2 is shown for the survival assays in c and e, full results and statistics for all trials are presented in Table S3. (f) Relative gene expression of stress regulating transcription factors in *rrf‐3(pk1426)* worms fed with EV or *pal‐1* (RNAi). The student's t‐test corrected with Holm‐Sidak multiple testing was used with *p < 0.05. (g) Relative expression of *elt‐2* and *elt‐3* mRNA levels under control, cadmium, or acrylamide exposure in *rrf‐3(pk1426)* worms fed with EV or *pal‐1* (RNAi). All bar graphs display means and error bars indicate a standard deviation of *N* = 4 samples. *p < 0.05, **p < 0.01, and ***p < 0.001 as determined by two‐way ANOVA with Holm‐Sidak post hoc tests in b, d, and g.

Next, we employed the Y2H system using a GAL4‐DBD‐CCF‐1 fusion protein to probe against a mixed‐stage *C. elegans* GAL4‐AD cDNA prey library to identify potential CCF‐1 binding partners. The Y2H screen revealed two consistent prey binding partners of CCF‐1 that corresponded to clones encoding the CCR4 and PAL‐1 proteins (Figure 5a, Figure S4). CCR4 encodes a catalytic subunit of the CCR4‐NOT complex and this protein has previously been shown to directly interact with CCF‐1 in *C. elegans* (Nousch et al., 2013). Meanwhile, PAL‐1 encodes the orthologue of the human caudal‐related homeobox 1 (CDX1) transcription factor with a characterized role in embryonic posterior patterning and male tail development in *C. elegans* (Edgar et al., 2001). To test if PAL‐1 co‐regulates *ccf‐1* dependent stress genes, we used RNAi to knockdown *pal‐1* and performed qPCR to quantify its effects on cadmium and acrylamide‐induced gene expressions. Knockdown of *pal‐1* in the N2 wildtype strain did not lead to substantial changes to cadmium and acrylamide‐induced gene expression (data not shown), perhaps due to incomplete penetrance of the *pal‐1* RNAi. Given that a null mutant is not available, we then repeated the experiment using the RNAi‐sensitive *rrf‐3(pk1426)* strain and found that *pal‐1* knockdown broadly reduced the activation of *ccf‐1* dependent cadmium genes. (Figure 5b). Knockdown of *pal‐1* in the *rrf‐3(pk1426)* strain also reduced cadmium survival compared to EV, suggesting a role for *pal‐1* in stress resistance (Figure 5c). Similarly, the knockdown of *pal‐1* in the *rrf‐3(pk1426)* worms treated with acrylamide attenuated the activation of *ccf‐1* dependent *gst* genes and reduced acrylamide survival (Figure 5d,e). To determine whether the reduced stress resistance was caused by potential pleiotropic effects stemming from initiating the RNAi knockdown starting at the L1 development stage, we knocked down *pal‐1* and *ccf‐1* in 1‐day‐old adults for 48 hours followed by exposure to cadmium and acrylamide (Figure S5a). Knockdown of both *pal‐1* and *ccf‐1* during adulthood reduced resistance to cadmium and acrylamide (Figure S5b,c), suggesting a post‐developmental requirement of both genes in mediating stress resistance.

Previous studies in *C. elegans* have implicated a role for *pal‐1* in embryogenesis through regulating the expression of key transcription factors such as HLH‐1 (Helix Loop Helix) that controls body wall muscle development (Baugh et al., 2005). To test the hypothesis that *pal‐1* may regulate the mRNA expression of stress‐responsive transcription factors, we knocked down *pal‐1* in the *rrf‐3(pk1426)* strain to determine its effect on nine stress‐responsive transcription factors including *skn‐1, hsf‐1, daf‐16, elt‐2* (erythroid‐like transcription factor)*, elt‐3*, *hizr‐1*, *nhr‐46* (nuclear hormone receptor), *nhr‐49*, and *hlh‐30* (An & Blackwell, 2003; Budovskaya et al., 2008; Goh et al., 2014; Lin et al., 2018; Morley & Morimoto, 2004; Shomer et al., 2019). Of the nine transcription factors, we found that the knockdown of *pal‐1* led to a substantial decrease in both *elt‐2* and *elt‐3* mRNA levels by 45% and 62% respectively (Figure 5f). This is consistent with a previous report demonstrating reduced *elt‐3* expression after *pal‐1* RNAi as determined by a fluorescent transcriptional reporter (Baugh et al., 2005). The *elt‐2* and *elt‐3* genes encode members of the GATA transcription factor; *elt‐2* functions in the intestine to regulate intestinal differentiation while *elt‐3* primarily regulates epidermal gene expression (Fukushige et al., 1998; Gilleard & McGhee, 2001). Both *elt‐2* and *elt‐3* have been shown to serve as a co‐factor to the SKN‐1 transcription factor, with *elt‐2* required for *skn‐1*‐dependent pathogen response and *elt‐3* required for *skn‐1*‐dependent oxidative stress response (Block et al., 2015; Hu et al., 2017). Consistently, depletion of *elt‐2* compromises *C. elegans* immunity and mutation to *elt‐3* decreases *C. elegans* resistance to oxidative stress (Block & Shapira, 2015; Budovskaya et al., 2008; Hu et al., 2017). We found that the knockdown of *pal‐1* also significantly reduced *elt‐2* and *elt‐3* mRNA expression under cadmium (−58%, −60%), and acrylamide (−53%, −65%) conditions in comparison to EV (Figure 5g). The 53% reduction in *elt‐2* mRNA under acrylamide condition was not statistically significant (*p* = 0.13), this is potentially due to the stringency of the two‐way ANOVA test used. In support of *elt‐2* and *elt‐3* as downstream targets of PAL‐1, we found that the PAL‐1 DNA binding motif 5′‐GYAATWAA‐3′ was present within the 5′ upstream region of both the *elt‐2* and *elt‐3* sequences (Figure S6a).

We next examined whether *ccf‐1* knockdown exerted a similar effect on *elt‐2* and *elt‐3* expression using our RNA sequencing data. Unlike *pal‐1* RNAi, the knockdown of *ccf‐1* led to an elevated basal level of *elt‐2* and *elt‐3* (Figure S6b,c), this is consistent with *ccf‐1*'s deadenylase activity where the loss of its function increases the poly‐A tail length and transcript stability (Figure 3b). However, under stress, we found that the knockdown of *ccf‐1* attenuated the relative‐fold activation of *elt‐2* and *elt‐3* mRNA compared to EV (Figure S6d,e). This suggests that *ccf‐1* is required for the activation of *elt‐2* and *elt‐3* in response to cadmium and acrylamide and these effects under stress are consistent with the patterns observed for *pal‐1* RNAi (Figure 5g).

Overall, these results show that PAL‐1 interacts with CCF‐1 protein, and that the knockdown of *pal‐1* reduces *C. elegans* survival to cadmium and acrylamide stress by potentially attenuating the mRNA expression of the *elt‐2* and *elt‐3* transcription factors that are required to mount a transcription response to environmental stress.

### 2.5 ELT‐2 acts downstream of *ccf‐1* in a stress and gene‐specific manner

**FIGURE 6:**
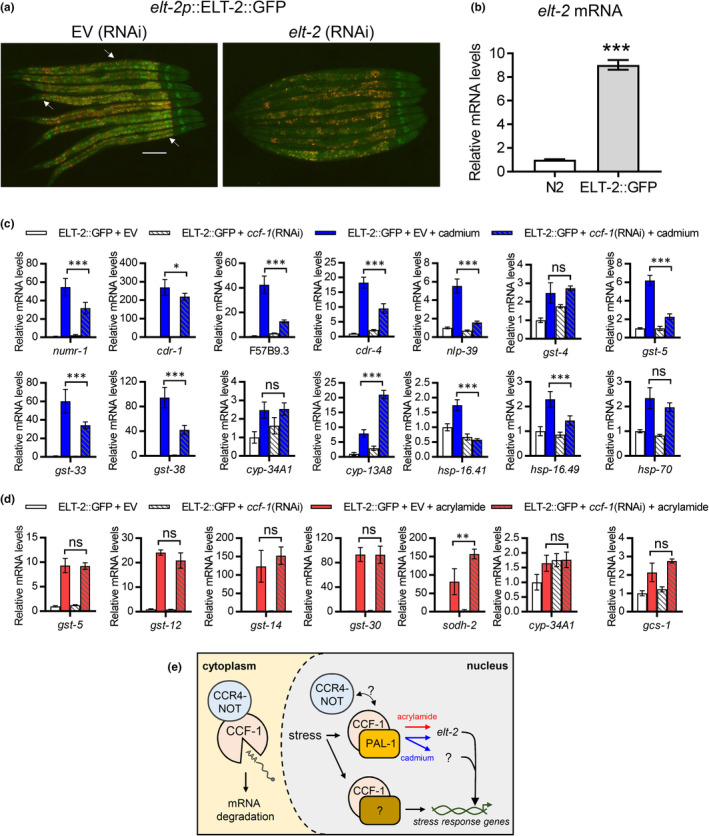
ETL‐2 functions are downstream of some *ccf‐1*‐dependent stress gene transcription. (a) Representative fluorescent micrograph of *elt‐2p*::ELT‐2::GFP fed with EV or *elt‐2* (RNAi). Arrow marks examples of the ELT‐2::GFP signal in the EV group, scale bar is 100 μm. (b) Relative mRNA expression of *elt‐2* in the ELT‐2::GFP strain. ***p < 0.001 as determined by student's *t*‐test. Relative mRNA expression of *ccf‐1* dependent (c) cadmium or (d) acrylamide inducible genes as identified from RNA sequencing measured via qPCR in the ELT‐2::GFP strain fed with EV or *ccf‐1* (RNAi) under control or stress conditions. *p < 0.05, **p < 0.01, and ***p < 0.001 as determined by two‐way ANOVA with Holm‐Sidak post hoc tests. All bar graphs display means and error bars indicate a standard deviation of *N* = 4 samples. (e) Proposed mechanism of CCF‐1 function in *C. elegans* stress response regulation. The CCR4‐NOT complex serves as a deadenylase complex in the cytoplasm. In response to stress, the CCF‐1 subunit together with the PAL‐1 transcription factor regulates the activation of stress genes through *elt‐2* under acrylamide and through *elt‐2* and other yet‐to‐be‐identified factor(s) under cadmium. It remains to be determined if the CCR4‐NOT complex as a whole is involved in the activation of stress genes and if factors other than PAL‐1 can interact with CCF‐1 to regulate this process.

To further investigate the relationship between *ccf‐1* and *elt‐2*, we created an integrated ELT‐2::GFP strain that stably overexpresses the ELT‐2 protein under its native promoter (Figure 6a,b). ELT‐2 was chosen as it serves as the primary co‐transcriptional regulator in the intestine, which is a major tissue of the *C. elegans* stress response. We chose several cadmium and acrylamide‐induced genes that were *ccf‐1* dependent from our RNA sequencing data and measured how these genes are regulated in the ELT‐2::GFP strain after *ccf‐1* RNAi. Knockdown of *ccf‐1* in ELT‐2::GFP attenuated the expression of most but not all cadmium‐responsive genes compared to EV. Notably, overexpression of ELT‐2 suppressed *ccf‐1*'s requirement for the expression of *gst‐4*, *cyp‐34A1*, *cyp‐13A8*, and *hsp‐70* under cadmium (Figure 6c). We then measured the effects of *ccf‐1* RNAi in ELT‐2::GFP under acrylamide, and surprisingly, we found that the knockdown of *ccf‐1* did not attenuate the expression of any of the seven genes tested (Figure 6d). These data suggest that *elt‐2* acts downstream of *ccf‐1* and that overexpression of ELT‐2 was sufficient to bypass the requirement of *ccf‐1* in the acrylamide stress response. Conversely, given that ELT‐2 overexpression was able to bypass the requirement of *ccf‐1* in some but not all cadmium‐inducible genes, it suggests that *elt‐2* is only partially required for the cadmium stress response that also involves additional factors downstream of *ccf‐1* (Figure 6e).

## 3 DISCUSSION

Transient activation of gene transcription provides a strategy for cells to adapt and respond to stressful conditions based on environmental demand and is critical for organismal survival. Under stress, transcription factors are recruited to gene promoters to initiate transcription based on the distress signal and the response required, and multiple transcription factors often function in parallel for a synergetic response. Here, we show that the *C. elegans* CCR4‐NOT subunit CCF‐1 physically interacts with the PAL‐1 transcription factor and both are required for the transcriptional activation of multiple classes of stress‐protective genes in response to cadmium and acrylamide. We propose that this mechanism of stress‐induced gene regulation in part signals through *elt‐2*, which encodes a transcriptional co‐regulator with demonstrated roles in multiple *C. elegans* stress responses (Block & Shapira, 2015). However, given that an epistasis analysis between *ccf‐1* and *pal‐1* was not performed due to the lack of a viable loss of function mutant for either gene, it is possible and likely that *ccf‐1* also functions with other transcription factors not identified in this study for environmental stress‐induced transcriptional regulation (Figure 6e).

### 3.1 CCR4‐NOT in stress response and transcription

A role for the CCR4‐NOT complex in stress response was initially demonstrated in yeast where CCR4‐NOT mutants showed increased sensitivity to replication stress and defects in accumulating *RNR* (ribonucleotide reductase) genes in response to DNA damage (Mulder et al., 2005). Mechanistically, it was later shown that CCR4‐NOT is recruited to the promoter and open reading frames of *RNR* genes to facilitate transcription initiation and elongation in response to DNA damage (Kruk et al., 2011). Genome‐wide cross‐linking analyses have also shown that CCR4‐NOT is predominantly recruited to SAGA‐regulated genes in yeast that typically encode highly inducible genes, suggesting a potential role for CCR4‐NOT in stress regulation in yeast (Venters et al., 2011). In human cells, CCR4 serves as a coactivator of several nuclear hormone receptors where CCR4 overexpression enhanced the activation of these receptors while CCR4 knockdown reduced receptor activation and decreased the abundance of hormone‐responsive target genes (Garapaty et al., 2008). In mice, the CCF‐1 homolog CNOT7 directly interacts with retinoid X receptor β to function as a transcriptional co‐activator and knockout of *Cnot7* impairs retinoic acid induced transcription (Nakamura et al., 2004). We show in this study that multiple subunits of the CCR4‐NOT complex positively promote the transcription of stress‐inducible genes in *C. elegans*. In this instance, the CCF‐1 subunit of the CCR4‐NOT complex interacts physically and functionally with the PAL‐1 transcription factor in promoting stress‐induced gene expression. Knockdown of *ccf‐1* compromises the oxidative stress resistance of *C. elegans* as evident by reduced survival to cadmium and acrylamide stress along with increased sensitivity to acrylamide‐induced dopaminergic neurodegeneration. As *ccf‐1* and *pal‐1* knockdown both affected a broad network of stress‐responsive gene classes, we propose this interaction likely indirectly promotes stress resistance by activating the expression of *elt‐2* and *elt‐3* that encode master transcriptional co‐regulators involved in multiple stress responses (Block & Shapira, 2015).

Given that a wide network of different stress‐responsive genes is affected by *ccf‐1* knockdown, an alternative possibility is that the CCR4‐NOT complex may regulate stress gene transcription through chromatin remodeling. Dynamic modification to nucleosome organization at promoters is a well‐conserved mechanism that governs the temporal expression of stress‐responsive genes (De Nadal et al., 2011). A recent study in yeast has shown that loss of *caf1* (yeast orthologue of *ccf‐1*) results in an increased transcript abundance and transcriptional efficiency in all heterochromatic regions, supporting a negative role for the CCR4‐NOT complex in promoting gene expression via transcriptional silencing to maintain a heterochromatin state (Monteagudo‐Mesas et al., 2022). This is in contrast to the finding of this study where we show that depletion of *ccf‐1* in *C. elegans* results in a decreased transcription of stress‐responsive genes, suggesting a positive role for the CCR4‐NOT complex in promoting gene expression under stress. It remains to be determined whether other transcription factors besides PAL‐1 interact with CCF‐1 to regulate this response and if CCF‐1 alone or the entire CCR4‐NOT complex is involved in transcriptional regulation. To the latter question, our results showed that knockdown of other CCR4‐NOT subunits including *ntl‐2*, *ntl‐3*, and *let‐711* can also attenuate activation of cadmium‐induced genes, suggesting the involvement of the CCR4‐NOT complex as a whole in the transcriptional stress response.

### 3.2 The *elt* transcriptional network

The GATA transcription factors *elt‐2* and *elt‐3* have both been shown to function with transcription factors in the *C. elegans* intestine and epidermis respectively to regulate the expression of a wide network of stress‐responsive genes. Following infection to *Pseudomonas aeruginosa*, ELT‐2 cooperates with the transcription factors SKN‐1 and ATF‐7 (Activating Transcription Factor) to promote the expression of different classes of immune genes (Block & Shapira, 2015). ELT‐2 functions as the predominant regulator of intestinal gene expression, which is the major site of pathogen and xenobiotic stress response in *C. elegans*. Consistently, direct gene targets of ELT‐2 predicted through SAGE analysis include those functioning in metal detoxification, cytochrome P450 metabolism, and glutathione activity (McGhee et al., 2009). Analogous to ELT‐2, ELT‐3 appears to also function as a stress transcriptional co‐regulator with the distinction that ELT‐3 primarily acts in the epidermis (Block & Shapira, 2015). In response to oxidative stress, ELT‐3 directly binds to the SKN‐1 transcription factor to promote the expression of *gst*‐related antioxidant genes (Hu et al., 2017). ChIP analysis indicates that one‐third of ELT‐3's targets are also bound directly by the SKN‐1 transcription factor, reinforcing the role of ELT‐3 as a co‐regulator of SKN‐1 (Block & Shapira, 2015). In response to pathogen infection and osmotic stress, ELT‐3 controls the expression of neuropeptide‐like protein genes potentially through a mechanism involving the STA‐2 (Signal Transducer and Activator) transcription factor (Dodd et al., 2018). Lastly, ELT‐3 is also required for the expression of DAF‐16‐regulated genes in the hypodermis, suggesting that it also functions within the insulin/IGF‐1 pathway (Zhang et al., 2013).

We have previously shown that oxidative stress promotes the nuclear localization of SKN‐1::GFP in both the intestine and the epidermis, and this is supported by the up‐regulation of the *gst‐4p*::GFP reporter in both tissues (Wu et al., 2016). Knockdown of *skn‐1* in either the intestine or epidermis alone reduces *C. elegans* resistance to oxidative stress, supporting the idea that SKN‐1 activity in both tissues is critical for oxidative stress resistance (Wu et al., 2016). Interestingly, our data show that overexpression of the intestinal transcriptional co‐factor ELT‐2 can suppress the requirement of *ccf‐1* in the transcription of acrylamide‐responsive genes tested. Given that majority of *ccf‐1*‐dependent acrylamide‐responsive genes function in xenobiotic detoxification which is known to be controlled by the SKN‐1 transcription factor, it is plausible that *ccf‐1* signals through ELT‐2 that cooperates with SKN‐1 in the intestine to promote the acrylamide‐induced transcriptional response. The overexpression of ELT‐2 did not bypass cadmium's requirement of *ccf‐1* for activation of all heavy metal response genes tested, this would suggest that additional factors besides ELT‐2 act downstream of *ccf‐1* in regulating the transcriptional response. A possibility is that some of the *ccf‐1* dependent cadmium genes tested may be expressed in tissues other than the intestine, and may require co‐activators such as ELT‐3 to facilitate stress‐responsive gene expression. We hope to address this in our future work to further explore how a common requirement for *ccf‐1* in multiple stress responses may signal through stress‐specific downstream mechanisms.

### 3.3 Role of *ccf‐1* in longevity

Genes that regulate stress response in *C. elegans* are often also factors that influence aging. Our results show that the knockdown of *ccf‐1* shortens normal lifespan and completely suppressed the longevity of the *skn‐1* gain of function mutant. This is consistent with our transcriptome analysis revealing that *ccf‐1* dependent stress genes highly cluster to *skn‐1* regulated glutathione metabolism. Interestingly in the *daf‐2* mutant where *daf‐16* is activated, the degree of lifespan extension compared to wildtype was similar when worms were fed with EV or *ccf‐1* RNAi. This could be interpreted as the mechanism through which *ccf‐1* knockdown shortens lifespan is independent of the downstream pathways through which the *daf‐2* mutant extends longevity. While this interpretation would conflict with evidence that *skn‐1* functions downstream of *daf‐2* mutant longevity, a possible explanation is that the knockdown of *ccf‐1* may not restrict other regulatory functions of *skn‐1* such as lipid metabolism or collagen gene expression that have been directly implicated in longevity regulation (Ewald et al., 2015; Steinbaugh et al., 2015). In support of a diminished role for *skn‐1* dependent xenobiotic genes in *daf‐2* mutant longevity, we previously showed that loss of function mutation to the *xrep‐4* (xenobiotics response pathways) gene that specifically attenuates *skn‐1* dependent *gst* gene expression does not reduce wildtype or *daf‐2(e1370)* lifespan (Wu et al., 2017). It is also possible that given the pleiotropic function of the CCR4‐NOT complex in mRNA regulation, its role in lifespan regulation may be linked to its deadenylase activity not explored in this study. Nonetheless, these data demonstrate a role for the CCR4‐NOT complex as a determinant of longevity and would be a subject of interest in future studies.

Overall, our study provides evidence that the CCR4‐NOT complex is a key regulator for the transcriptional response to various environmental stressors in *C. elegans* and highlights its role in organismal longevity and stress resistance. This finding further strengthens our collective knowledge of the eukaryotic CCR4‐NOT complex with demonstrated roles ranging from transcription initiation and elongation to mRNA decay.

## 4 MATERIALS AND METHODS

### 4.1 *C. elegans* strains

The following strains were used: N2 Bristol wildtype, QV151 *qvIs4[numr‐1p::GFP]*, BZ555 *egIs1[dat‐1p::GFP]*, VP198 *kbIs5[gpdh‐1p::GFP; rol‐6(su1006)]*, QV212 *skn‐1(k1023)*, AGD710 *uthIs235 [sur‐5p::hsf‐1::unc‐54 3′UTR; myo‐2p::tdTomato::unc‐54 3′ UTR]*, CB1370 *daf‐2(e1370) III*, MWU110 *cwwIs4 [ccf‐1p::ccf‐1::GFP::unc‐54 3’UTR; myo‐2p::tdTomato::unc‐54 3′ UTR]*, NL2099 *rrf‐3(pk1426) II,* MWU205 *cwwIs13[elt‐2p::elt‐2::GFP + unc‐119(+)]*. MWU205 was created by UV integration of SD1963 *unc‐119(ed3) III; rde‐1(ne300); gaEx234 [elt‐2p::elt‐2::GFP + unc‐119(+)] V*, followed by six rounds of outcross to remove potential background mutation accrued from UV integration and the *rde‐1(ne300)* mutation. It was not determined whether the *unc‐119(ed3) III* allele is still present in MWU205. All *C. elegans* strains were cultured at 20°C using standard methods (Brenner, 1974), with the exception of *daf‐2(e1370)* and *rrf‐3(pk1426)* which were grown at 16°C during development followed by a shift to 20°C on the first day of adulthood.

### 4.2 Genome‐wide RNAi screen and RNAi experiments

RNAi screen was performed as previously described in detail (Wu et al., 2019). Briefly, synchronized L1 QV151 larvae were obtained using hypochlorite treatment and grown in liquid nematode growth media (NGM) and fed with dsRNA producing HT115(DE3) bacteria for 2 days, followed by exposure to 100 μM cadmium chloride for 24 h and screened for suppression of *numr‐1p*::GFP fluorescence. Approximately 19,000 dsRNA clones from the MRC genomic RNAi feeding library (Geneservice) and the ORFeome RNAi feeding library (Open Biosystems) were screened. Clones that suppressed *numr‐1p*::GFP fluorescence were rescreened three additional times for validation. For all other RNAi experiments in this study, NGM agar plates containing 50 μg mL^−1^ carbenicillin and 100 μg mL^−1^ of isopropyl β‐d‐thiogalactopyranoside (IPTG) were used. *E. coli* expressing the pPD129.36 (L4440) plasmid was used as an RNAi control and referred to as empty vector (EV) in this study as this plasmid encodes 202 bases of dsRNA not homologous to any *C. elegans* genes. For the *pal‐1* RNAi experiments, the enhanced RNAi‐sensitive strain *rrf‐3 (pk1426)* was used.

### 4.3 Microscopy and fluorescent analysis

To analyze *numr‐1p*::GFP and *gpdh‐1p*::GFP transcriptional reporters or the CCF‐1::GFP translation reporter, synchronized worms were grown on EV or *ccf‐1* RNAi‐seeded NGM agar plates until day 1 of adulthood followed by transfer to NGM agar plates seeded with corresponding RNAi containing 100 μM of cadmium for *numr‐1p*::GFP, 300 mM NaCl for *gpdh‐1p*::GFP, and 100 μM of cadmium or 5 mM of acrylamide for CCF‐1::GFP analysis. After 24 hours, worms were mounted on a glass slide containing a 2% agarose pad and immobilized with 2% sodium azide before imaging with a Zeiss Axioskop 50 microscope mounted with a Retiga R3 camera. Eight worms were mounted per slide and relative fluorescence was calculated using the Measure function in ImageJ followed by background subtraction. The background signal for each image was calculated by defining an area on the same image where the fluorescence signal was absent, with the dimension of the background area subtracted constant for all images. For the ELT‐2::GFP reporter, synchronized L1 worms fed with EV or *elt‐2* RNAi after 48 hours were imaged using the methods described above. Images were taken with a GFP filter as well as a DAPI filter to create a merged composite image to help differentiate between the ELT‐2::GFP signal and intestine autofluorescence.

Methods used to assess acrylamide‐induced dopaminergic degeneration via *dat‐1p*::GFP were described previously in detail (Murray et al., 2020). Briefly, synchronized L1 *C. elegans* were grown on NGM agar plates seeded with EV or *ccf‐1* RNAi until day 1 of adulthood followed by transfer to NGM agar plates containing 5 mM of acrylamide seeded with the corresponding RNAi bacteria and grown until 6 days old. Adult worms were separated from their progeny via picking with a sterilized metal pick onto fresh acrylamide agar plates during the reproductive window. On day 6, worms were prepared for imaging using the procedures described above. Scoring criteria for dopaminergic integrity were as follows, wildtype indicates smooth and continuous cephalic neuron (CEP) sensilla dendrite located at the anterior portion of the worm, blebbing indicates at least 1 abnormal punctae along the CEP dendrites, and breaks indicate discontinuation within the CEP dendrite. Three trials were performed with *N* = 15 worms scored per condition for each trial. All grayscale images were converted to color using ImageJ, with composite images displaying both GFP and RFP/DAPI colors created using the Merge Channel function when applicable.

### 4.4 Lifespan and survival assays

All assays were performed at 20°C using methods previously described (Wu et al., 2019), except for *daf‐2(e1370)* and *rrf‐3(pk1426)* which were grown at 16°C during development. For lifespan assays, synchronized L1 worms obtained through the hypochlorite treatment were grown on the appropriate RNAi‐seeded NGM agar plates until adulthood. One‐day‐old adult worms were moved to new plates and continuously transferred via daily picking during the reproductive window to accomplish progeny separation. For survival assays, synchronized L1 worms were first grown on control NGM plates seeded with appropriate RNAi bacteria until the first day of adulthood, followed by transfer to NGM plates containing either 100 μM of cadmium chloride or 5 mM of acrylamide seeded with the corresponding RNAi bacteria. For both lifespan and survival assays, the first day of adulthood is considered as 1‐day‐old, and worms were scored every 1–2 days for death by gentle prodding with a flame‐sterilized metal pick. Worms that do not respond to gentle prodding were considered dead, and worms with protruding vulva or gonad were recorded as censors. Three independent trials were formed for all assays with the number of animals scored in each condition and experiment listed in Table S3.

### 4.5 RNA extraction and qPCR

The Purelink RNA mini kit (12183020; ThermoFisher) was used to isolate total RNA with a Qsonica Q55 sonicator used for tissue lysis. Synchronized L1 N2 or *rrf‐3(pk1426)* worms were grown on NGM agar plates seeded with EV or corresponding RNAi until day 1 of adulthood followed by transfer to either control agar plates, agar plates containing 100 μM of cadmium chloride, or agar plates containing 5 mM of acrylamide, each seeded with the corresponding RNAi bacteria for an additional 24 h followed by RNA extraction. For each condition, *N* = 4 biological RNA replicates were prepared with each replicate containing ~500 worms. For qPCR, RNA was first treated with DNaseI (EN0521; ThermoFisher) followed by cDNA synthesis with the Invitrogen Multiscribe™ reverse transcriptase (4311235; ThermoFisher) using an Applied Biosystems ProFlex thermocycler. Real‐time PCR (qPCR) was performed with the PowerUp™ SYBR™ Green Master Mix (#A25741) in a QuantStudio3 system. Relative gene expression was normalized to the housekeeping gene *rpl‐2* (ribosomal protein large subunit) and *cdc‐42* (cell division cycle related), primers used for this study are shown in Table S4.

### 4.6 RNA‐sequencing and data analysis

Wildype N2 worms synchronized at the L1 stage were grown on NGM agar plates seeded with EV or *ccf‐1* RNAi until day 1 of adulthood followed by transfer to either control agar plates, agar plates containing 100 μM of cadmium chloride, or agar plates containing 5 mM of acrylamide, each seeded with the corresponding EV or *ccf‐1* RNAi for 24 h followed by RNA extraction. Total RNA from three biological replicates for each experimental group was extracted using the methods described above, with the exception that ~2000 to 3000 worms were used for each replicate. RNA samples were then sent to Novogen on dry ice for cDNA library construction with oligo(dT) enrichment and sequencing. Sequence annotation and data analysis were performed by Novogene. Correlation analysis between all sequenced samples is shown in Figure S7. Given that RNA was extracted from 1‐day‐old adults, a potential limitation is the mixing of RNA from fertilized embryos found across all samples that may contribute to the pool of sequenced adult worm RNA.

### 4.7 Y2H library screen

The full‐length *ccf‐1* cDNA clone was generated through PCR using the Q5® High‐Fidelity DNA polymerase (M0491L; New England BioLabs) and cloned into the pDEST32 vector containing the GAL4 DNA binding domain (DBD) through Gateway cloning (11789020 and 12538120; ThermoFisher) and used as the Y2H bait construct. A Y2H prey cDNA library of *C. elegans* genes was cloned into the pDEST22 vector containing the GAL4 activating domain (AD) using the CloneMiner™ cDNA library construction kit (A11180; ThermoFisher). A forward Y2H library screen was performed using the ProQuest Two‐Hybrid System (PQ1000101; ThermoFisher) to identify protein interactors of the *C. elegans* CCF‐1 protein.

Yeast colonies containing bait and prey constructs that grew on Trp^−^/Leu^−^/His^−^ + 25 mM 3‐Amino‐1,2,4‐triazole (3AT) selection plates were extracted using the Zymoprep Yeast Plasmid Miniprep II (Zymo Research, D2004) followed by Sanger sequencing for gene identification. Three independent colonies from each bait‐interacting prey construct were further tested for its interaction with CCF‐1 by evaluating for (1) growth on Trp^−^/Leu^−^/Ura^−^ selection plate, (2) negative growth on Trp^−^/Leu^−^ + 0.2% 5‐fluoroorotic acid selection plate, (3) growth on Trp^−^/Leu^−^/His^−^ + 25 mM AT selection plate, and (4) positive phenotype from the X‐gal assay. Yeast cells were normalized to OD_600_ = 0.5 for the 10^0^ concentration followed by serial dilution on the growth assay. Prey construct that passed three out of four selection tests was considered a positive CCF‐1 interacting protein. Yeast cells were imaged on a Bio‐Rad Gel Doc EQ system.

### 4.8 Statistical analyses

Graphical data and statistical analysis were performed using Graphpad Prism software (V7.04). Student's t‐test was used for the comparison of two groups with the Holm‐Sidak multiple test correction applied when the test is repeated for multiple genes, one‐way ANOVA with Dunnett's test was used for comparison of more than two groups, two‐way ANOVA with Holm‐Sidak test was used for comparison of two factors, and categorical data were analyzed using the Chi‐square test. Lifespan and survival data were analyzed with the log‐rank test using the OASIS2 software (https://sbi.postech.ac.kr/oasis2/history/). For RNA sequencing data, false discovery rate (FDR) correction was applied to determine the statistical significance. For all statistical tests, the following designations were used to indicate the *p*‐value, *p < 0.05, **p < 0.01, ***p < 0.001.

## AUTHOR CONTRIBUTIONS

Hadi Tabarraei, Brandon M. Waddell, Kelly Raymond, and Sydney M. Murray performed experiments and analyzed the results. Keith P. Choe helped conceive and design the RNAi screen; Ying Wang and Keith P. Choe generated the Y2H cDNA library. Cheng‐Wei Wu designed the study, performed experiments, analyzed results, and wrote the manuscript.

## CONFLICT OF INTEREST STATEMENT

The authors have no conflict of interest to declare.

## Supporting information


Figure S1

Figure S2

Figure S3

Figure S4

Figure S5

Figure S6

Figure S7

Table S1

## Data Availability

All datasets supporting this manuscript are found within the article and its [Link], [Link], [Link], [Link], [Link], [Link], [Link], [Link]. RNA‐sequencing data generated from this study (raw and annotated) are available on the NCBI GEO data repository GSE194057. Strains are available upon request.
