# Supplementary figures and images for: CCR4‐NOT subunit CCF‐1/CNOT7 promotes transcriptional activation to multiple stress responses in Caenorhabditis elegans
Source: Aging Cell. 2023 Feb 16;22(4):e13795. doi: 10.1111/acel.13795 (PMC10086529; doi:10.1111/acel.13795)

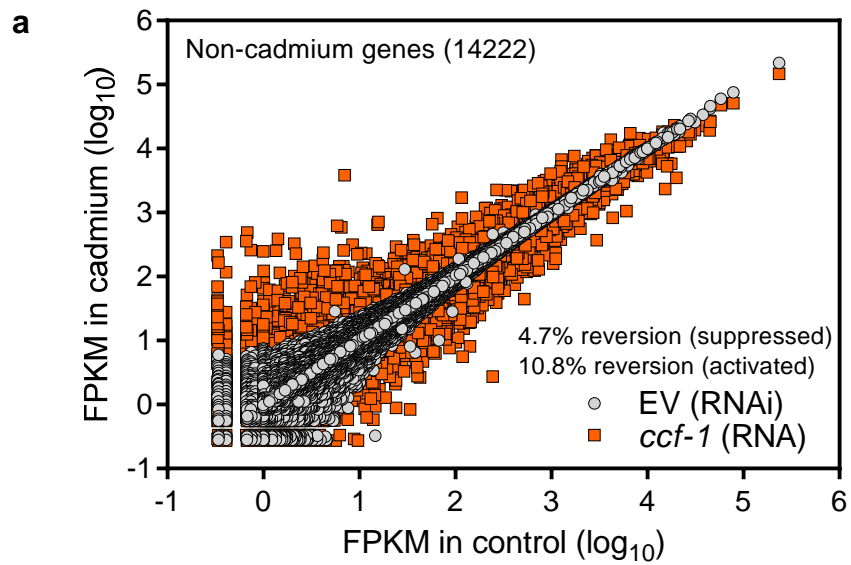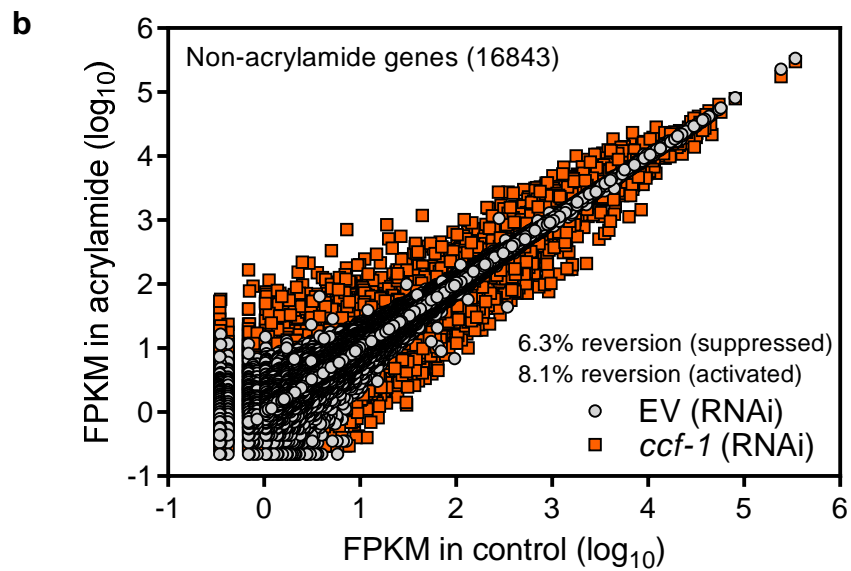

**c**

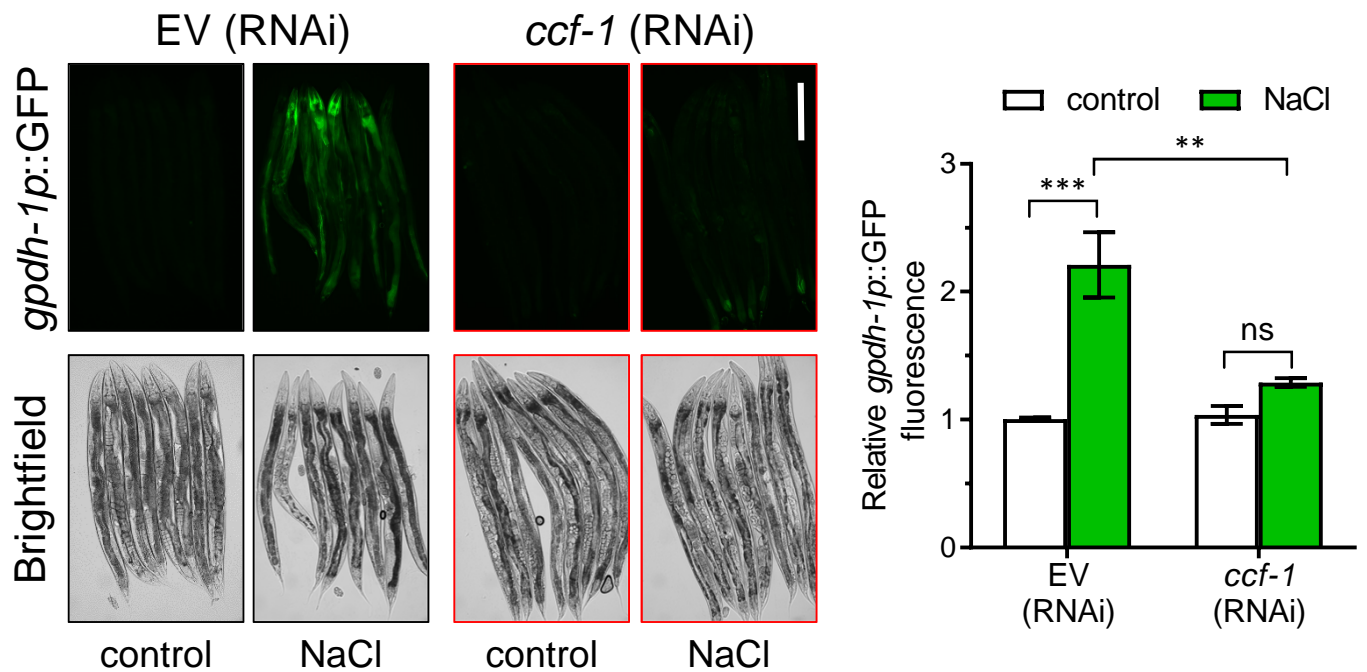

Supplement: Supplementary file 1 — Figure S1 [file ACEL-22-e13795-s005.pdf]

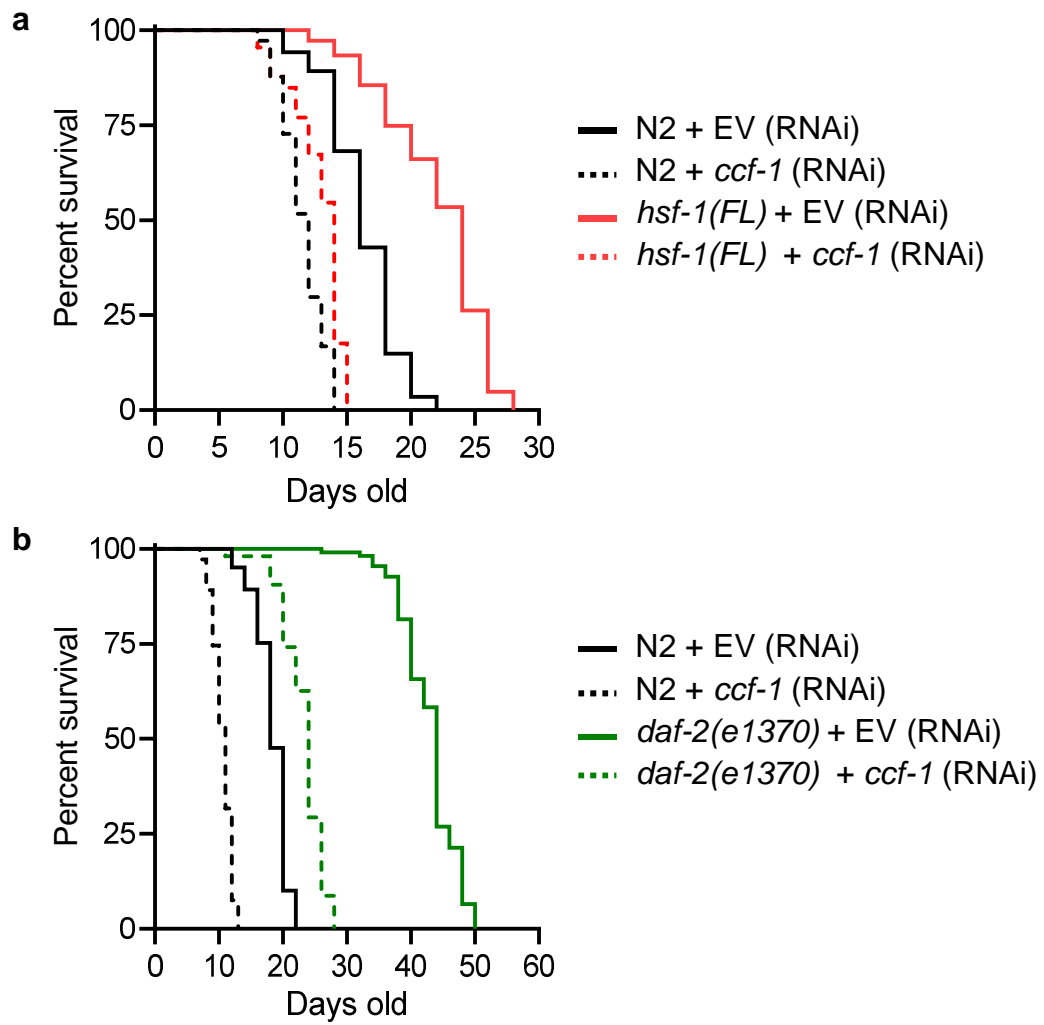

Supplement: Supplementary file 2 — Figure S2 [file ACEL-22-e13795-s002.pdf]

*ccf-1p::CCF-1::GFP*

**a**

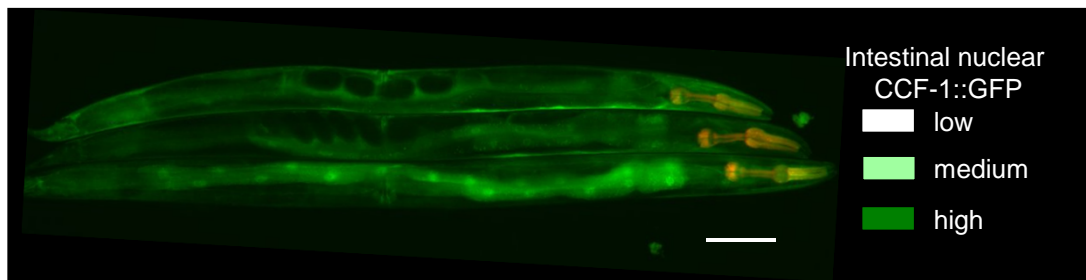

**b**

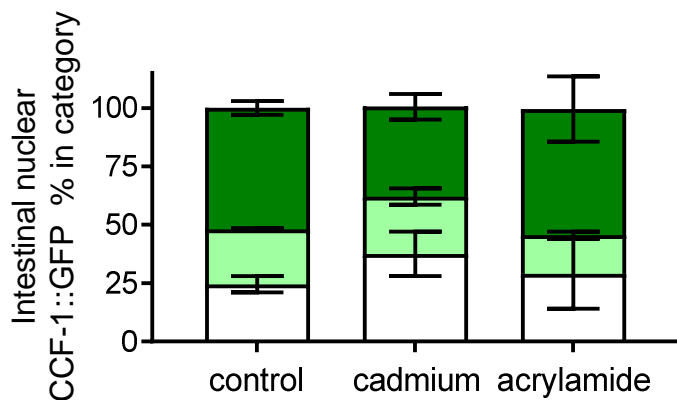

**c**

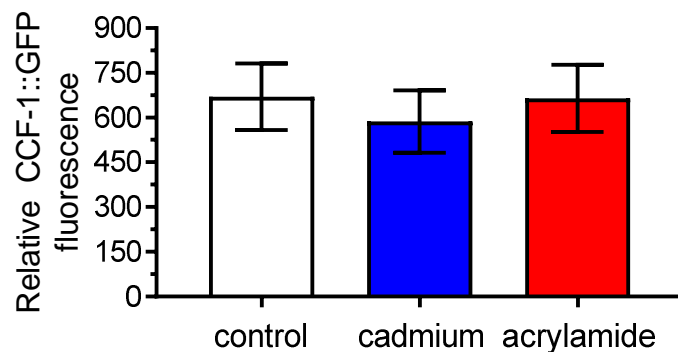

**d**

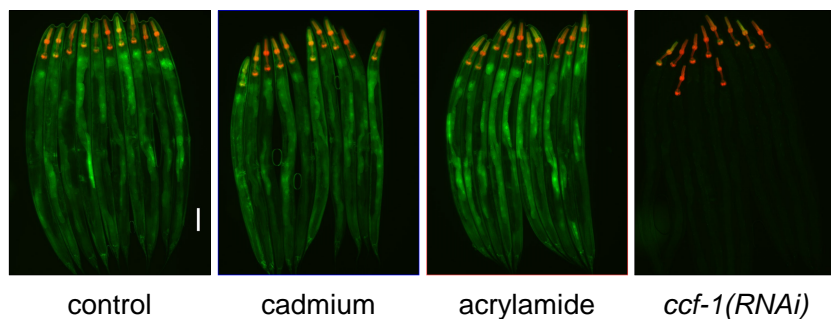

**e**

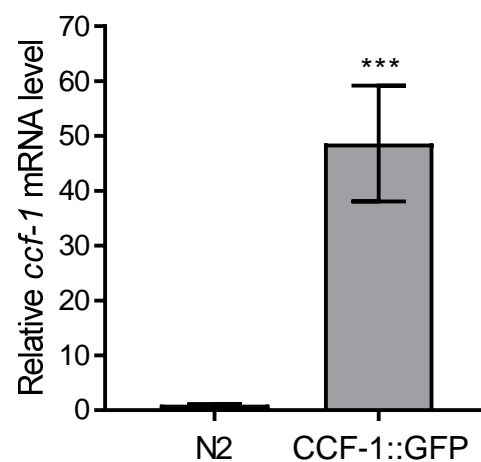

**f**

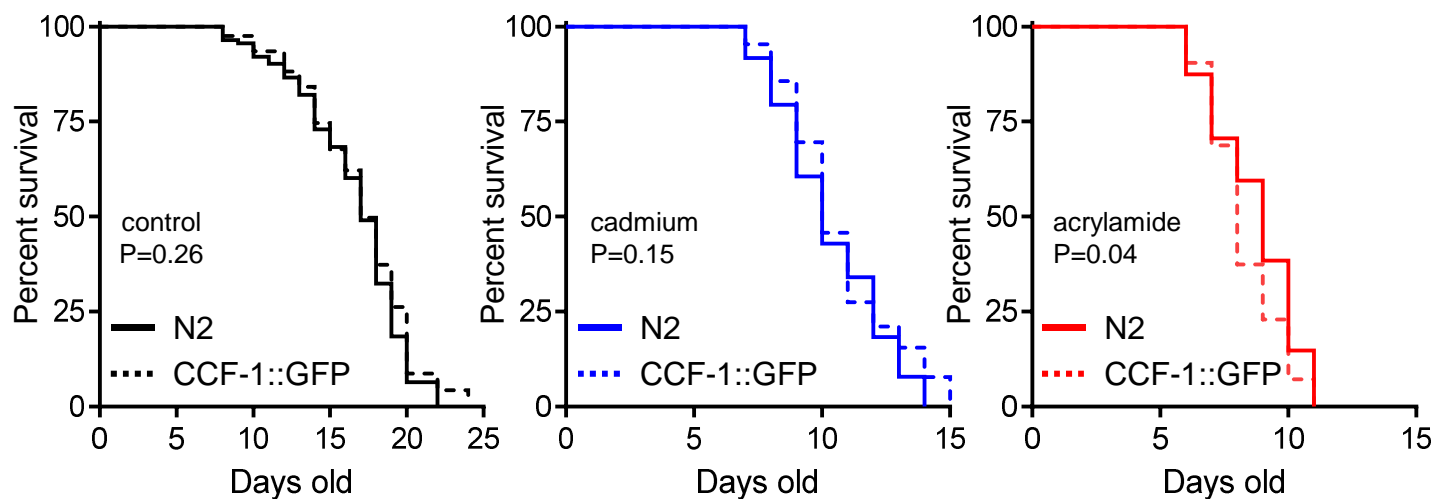

Supplement: Supplementary file 3 — Figure S3 [file ACEL-22-e13795-s007.pdf]

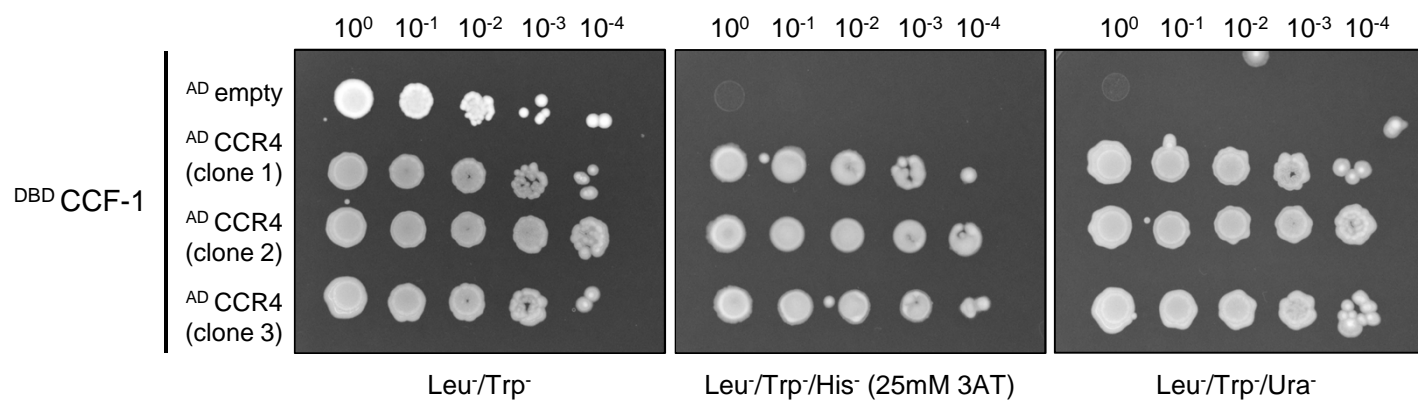

Supplement: Supplementary file 4 — Figure S4 [file ACEL-22-e13795-s003.pdf]

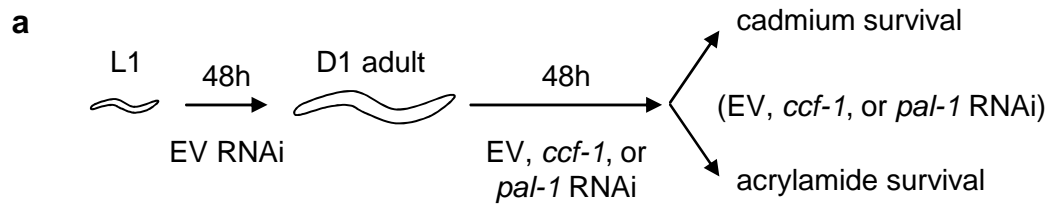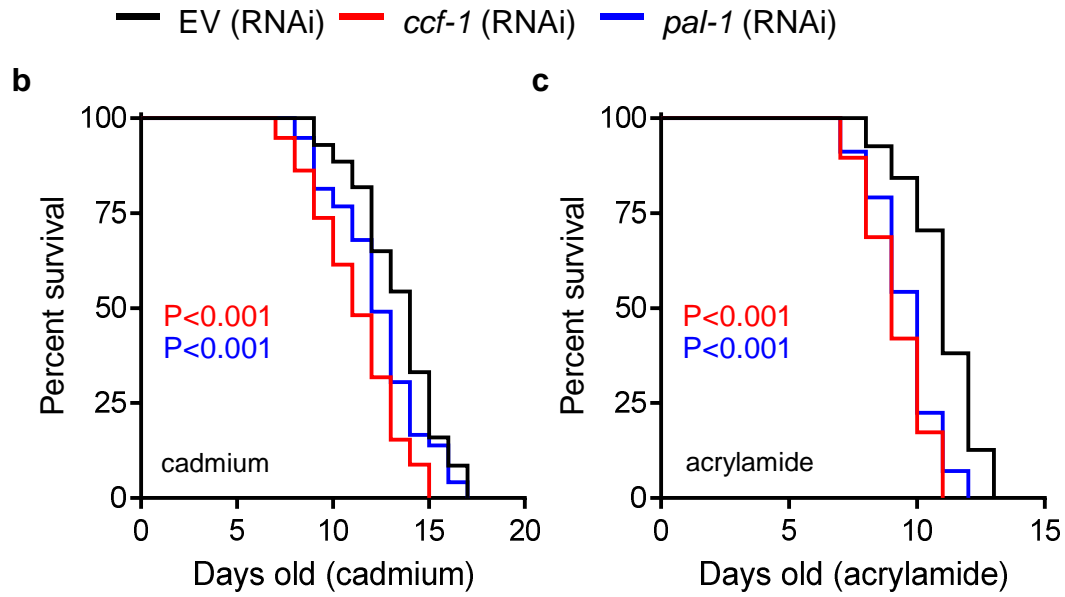

Supplement: Supplementary file 5 — Figure S5 [file ACEL-22-e13795-s004.pdf]

**a**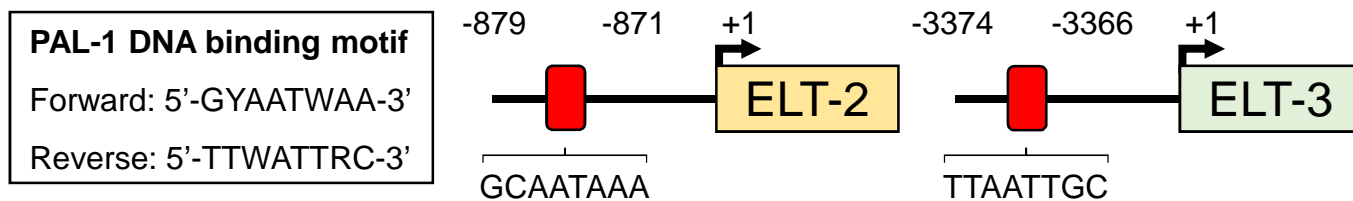**b**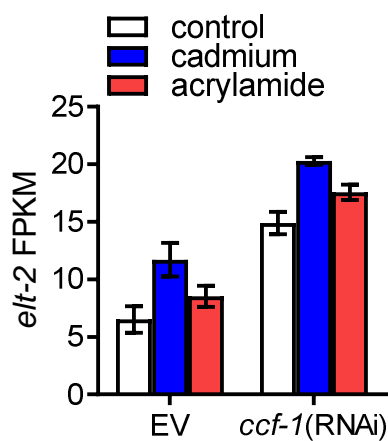**c**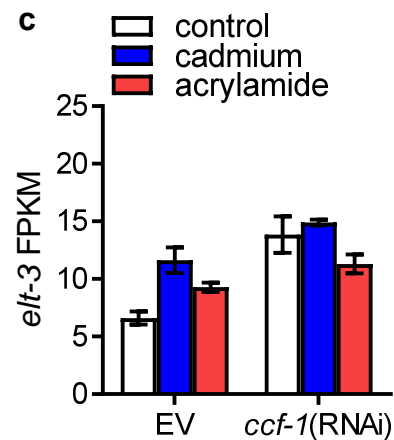**d**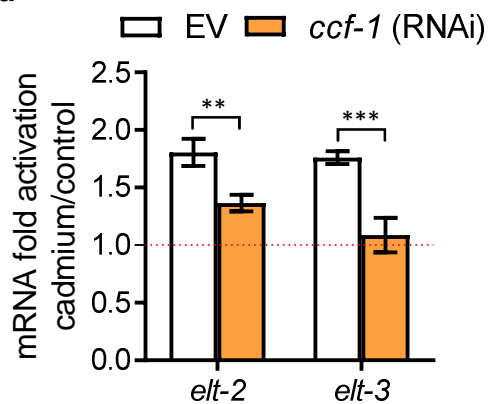**e**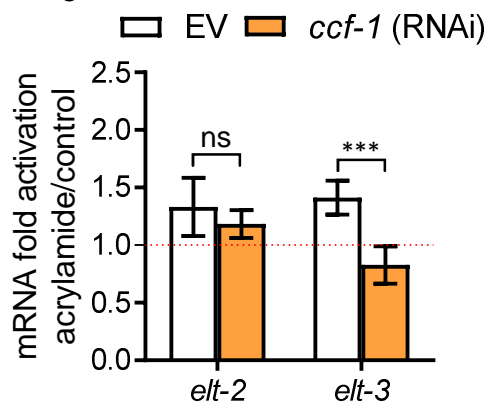

Supplement: Supplementary file 6 — Figure S6 [file ACEL-22-e13795-s008.pdf]

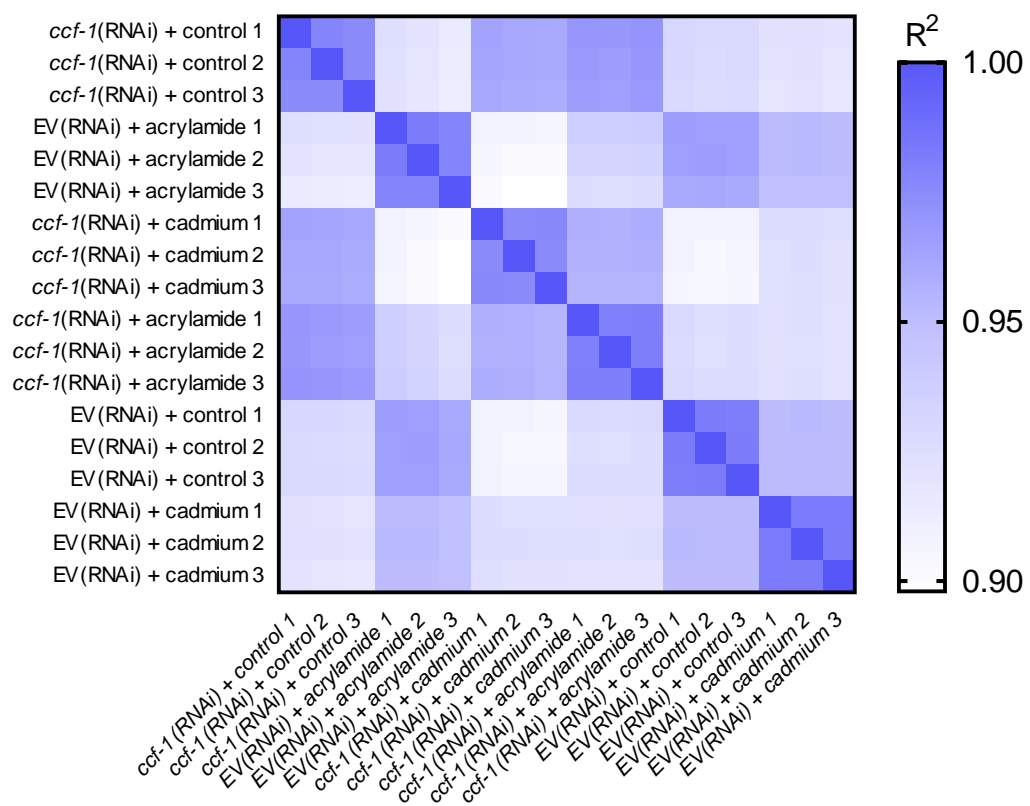

Supplement: Supplementary file 7 — Figure S7 [file ACEL-22-e13795-s006.pdf]
